# Effect of Si, Mn, Be and Sr Addition on the Tensile Properties of 6061 Type Alloys: Role of Aging Treatment

**DOI:** 10.3390/ma16031110

**Published:** 2023-01-27

**Authors:** Agnes M. Samuel, Ehab A. Elsharkawi, Mohamed H. Abdelaziz, Ehab Samuel, Fawzy H. Samuel

**Affiliations:** 1 Département des Sciences Appliquées, Université du Québec à Chicoutimi, Chicoutimi, QC G7H 2B1, Canada; 2 Division of Engineering, Saint Mary’s University, Halifax, NS B3H 3C3, Canada; 3 Département PEC, Université Française d’Égypte, Ville Shorouk, Le Caire 4923116, Egypt

**Keywords:** aluminum alloys, additives, intermetallics, tensile properties, age hardening

## Abstract

The present study was performed on a 6061-type alloy to examine the effects of minor additions (Si, Mn, Be, Sr) of the type of precipitated Fe-based intermetallics, in terms of Fe/Si ratios. All alloys were grain refined (0.15%Ti in the form of Al-5%Ti-1%B) to minimize hot tearing during casting. The effect of these intermetallics on the alloy tensile properties was also investigated. Tensile test bars were solutionized at 520 °C followed by quenching in warm water at 60 °C to avoid cracking. The quenched bars were aged at 175 °C for periods up to 100 h. Characterization of the formed intermetallics as well as phase precipitation were carried out using field emission scanning electron microscopy. In Be-treated alloys, α-Al_8_Fe_2_SiBe phase may precipitate along with α-Al_15_(Fe, Mn)_3_Si_2_ phase. In addition, Be results in fragmentation of the α-Fe phase when the alloy was Sr-modified, leading to better tensile properties, compared to those obtained from the base alloy under same conditions. It should be noted that this study does not promote the use of Be as it is a toxic element.

## 1. Introduction

Aluminum 6061 alloy is widely used in the aircraft and aerospace industries. This category of lightweight alloys is heat-treatable to produce high strength. It has been suggested by several authors that the precipitation that occurs during aging of Al-Si-Mg alloys is the decomposition of the supersaturated aluminum matrix to GP-1 and GP-2 zones (mostly β″ needles) followed by precipitation of Mg_2_Si (β′ rods) and finally Mg_2_Si (β platelets) [1,2,3,4,5,6,7].

Among the elements used to enhance the alloy strength in the T6 condition are magnesium (Mg), silicon (Si) and copper (Cu) [8]. According to Nowotink [7], the strength of 6xxx alloys following a T6 heat treatment is mainly linked with the volume fraction of β″ which mainly depends on the concentration of Mg and Si in the used alloy. In his work, the author reported that good tensile strength may be obtained from 6061 containing a high percentage of Mg, Si, Mn and iron (Fe). Pitchayyapillai et al. [9] examined the effect of silver (Ag) addition to 6061 alloy. Their results revealed that addition of (1–2%) Ag can be used instead of the current 6061 alloy due to its better performance and longer working life.

The effect of heat treatment on the properties of 6061 alloy was investigated by Hussein et al. [10]. Their findings showed that a heat-treated 2024 alloy would yield better mechanical properties than those offered by 6061 alloy. Mohamed et al. [11] recommended the application of high-pressure torsion to produce 6061 alloy with ultra-fine grain size (about 200 nm) and fine precipitation through the aging process. Thus, total hardness is attributed to the Hall-Petch relationship plus β′ precipitation. This technique is expected to increase the strength to 400 MPa with some ductility. The aging behavior of ultrafine-grained 6061 alloy processed through multi-directional forging was found to result in significant increase in the alloy work hardening as well as its ductility. The observed improvement was related to the evolution of a homogeneous microstructure with an ultrafine grain size (about 250 nm) [12].

Wang et al. [13] analyzed the microstructure of 6061 alloy after casting and diffusion annealing. Their findings indicate that applying different diffusion annealing treatments could result in phase change in the surface and the center of the cast piece. Void growth in 6061 alloy was studied by Agarwal et al. [14]. The void growth was analyzed quantitatively as a function of the stress state in notch tensile test specimens. Manganese concentration in 6xxx alloys above 0.5% would markedly increase both the ultimate tensile strength (UTS) and yield strength (YS) without reduction in the alloy ductility [15]. Farshidi et al. [16] reported that natural aging of aluminum 6061 alloy that was exposed to severe plastic deformation produced moderate recovery due to progression of the cell microstructure.

Beryllium (Be) is also used in aluminum alloys containing magnesium to reduce oxidation at elevated temperatures. Up to 0.1% Be is used in aluminizing baths for steel to improve adhesion of the aluminum film and restrict the formation of the deleterious iron-aluminum complex [17].

Elsharkawi et al. [18] and Ibrahim et al. [19] carried out extensive studies on the effect of Be addition on the tensile and impact properties of 356 and 357 alloys, popular commercial industrial alloys. The mechanical properties of Be-containing alloy improved significantly due to the alternation of Fe phase shape as well as fragmentation of β-Al_5_SiFe platelets. The present contribution serves to extend the work in terms of fragmentation of Fe-based intermetallics and the extent in the improvement in the alloy tensile properties to include 6061 alloys, due to their high industrial potential [20].

## 2. Experimental Procedures

Experiments were previously conducted to investigate the effect of using different coolant methods during drilling of aluminum 6061 (Al-0.65% Si-0.17% Fe-0.69% Mg) alloy plates. In order to understand the effect of aging treatment on the tensile properties of the alloy, the plates were melted in a 20 kg SiC crucible using an electrical resistance furnace. The melting temperature was held at 735 °C ± 5 °C. At this temperature, the melt was treated as follows: measured amounts of Sr (in the form Al-10% Sr master alloy), Mn (Al-25% Mn master alloy), Si (in the form of 99.94% pure metal) and Be (Al-5%Be master alloy) were added with the help of a perforated graphite bell under ventilation. The molten metal was degassed for ~30 min using pure argon (injected into the molten metal using a graphite impeller rotating at −150 rpm); the melt surface was thoroughly skimmed before pouring. In all cases, the hydrogen level was less than 0.1 mL/100 g Al (as measured by an AlScan™ apparatus (ABB Inc, Saint-Laurent, QC, Canada). The chemical analysis was carried out using a Spectrolab-JrCCD Spark Analyzer (SPECTRO Analytical Instruments Inc., Mahwah, NJ, U.S.A.). The average chemical compositions (three burns per alloy sample) are reported in Table 1. The molten metal was poured into a Stahl permanent mold (type ASTM B-108) heated at 450 °C. The estimated solidification rate was about 8 °C/s.

The solution heat-treated condition (coded T4) comprised of a solution treatment of 8 h at 520 °C in a forced-air furnace followed by quenching in hot water (60 °C). The quenched test bars were kept at −20 °C to prevent any natural aging. For artificial aging, the test bars were aged at 175 °C for times of 1, 3, 8, 24 and l00 h in the forced-air furnace, followed by air cooling. The test bars (five test bars per treatment/condition) were pulled to fracture at room temperature in an Instron^®^ Universal testing machine (INSTRON^®^, Norwood, MA, U.S.A.), at a strain rate of 4 × l0^−4^ s^−1^. A strain gauge extensometer (2-in or 5 cm range) was attached to the gauge section of the test bars for measuring the alloy ductility. Mechanical properties, namely, yield strength (YS) at 0.2% offset strain, ultimate tensile strength (UTS), and elongation to fracture (El%), were derived from the data acquisition system of the Instron machine.

In addition, thermal analysis was carried out using a 2 kg capacity SiC crucible heated in an electrical furnace. The molten metal (750 °C) was poured into a graphite mold heated at 600 °C and attached to a data acquisition system. A type-K thermocouple was placed from the bottom of the graphite mold up to half of its height. The total weight of the casting was about 700 g and the solidification rate was estimated as 0.8 °C/s [19].

A Hitachi-SU8000 field-emission scanning electron microscope (FESEM) (Hitachi High-Technologies Corporation, Tokyo, Japan), was employed to provide high-resolution images using a voltage as low as 5 kV, with an image resolution of 2.1 nm at 1 kV and 1.5 nm at 15 kV. The FESEM was equipped with energy dispersive X-ray spectrometer (EDS) and wavelength dispersive spectrometer (WDS) facilities.

## 3. Results and Discussion

### 3.1. General Remarks

**Figure 1:**
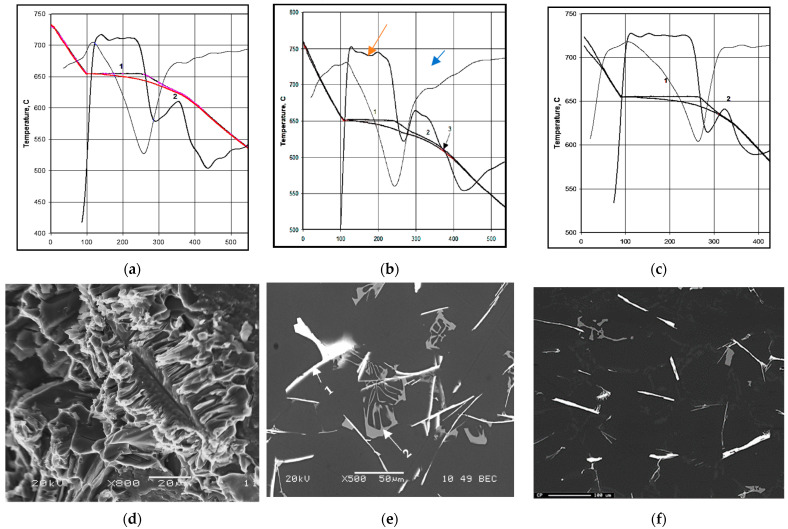
(**a**–**c**) Solidification curves and their first derivatives obtained from thermal analysis showing: (**a**) 1: development of α-Al network (655–653 °C), 2: Liq = Al + α-Fe at 633–611 °C (Fe/Si ratio 2.1); (**b**) 1: development of α-Al network (651–649 °C), 2: Liq = Al + α-Fe at 634–625 °C, 3: Liq + α-Fe = Al + α-Fe + β-Fe at 614–600 °C (Fe/Si ratio 1.6); (**c**) 1: development of α-Al network (655–652 °C), 2: Liq = Al + β-Fe, 3: liq + α-Fe = Al + β(614–600 °C) (Fe/Si = 1.6); (**d**) distribution of of Fe in α-Al_15_(Fe, Mn)_3_Si_2_ phase; (**e**) α-Fe (1) and β-Fe (2) phases; (**f**) distribution of Fe in β-Al_5_FeSi. Orange arrow = solidification curve; blue arrow = first derivative curve.

Khalifa et al. [21,22] investigated Fe-based intermetallics in the Al-Fe-Si ternary system. Their findings are summarized in Figure 1. In the absence of Mn or other neutralizer elements, the type of Fe-phase precipitated depends mainly on the Si/Fe ratio going from 1 (β-Al_5_FeSi) to 2 (α-Al_15_Fe_3_Si_2_). In order to elaborate on this aspect, a set of castings with different Si/Fe ratios, Sr concentration, and solidification rates is prepared and examined using image analysis technique.

### 3.2. Tensile Properties and Microstructural Characterization

**Figure 2:**
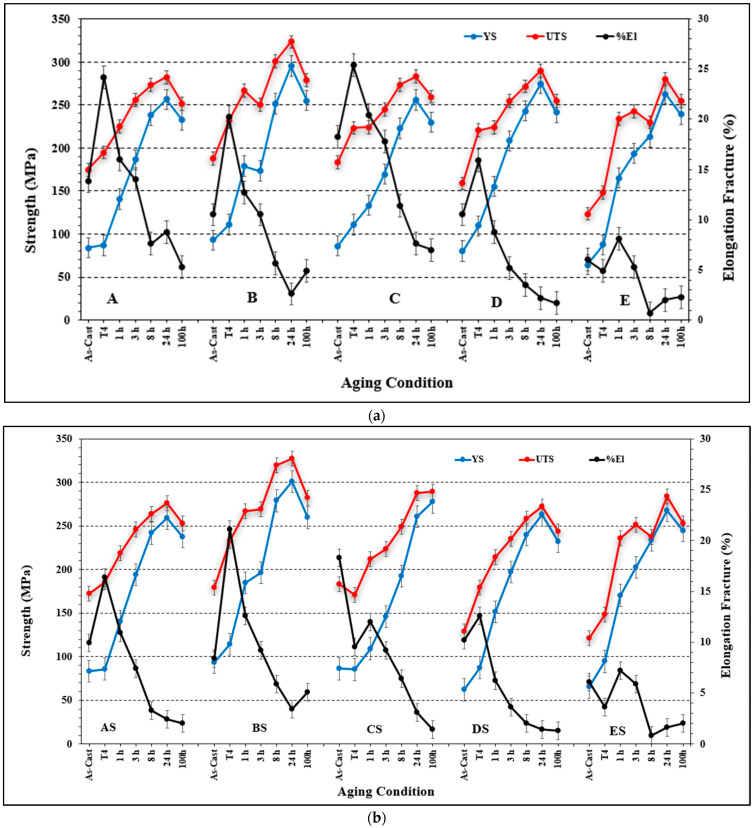
Effect of additives on the alloy tensile properties: (**a**) non-modified, (**b**) Sr- modified alloys.

**Figure 3:**
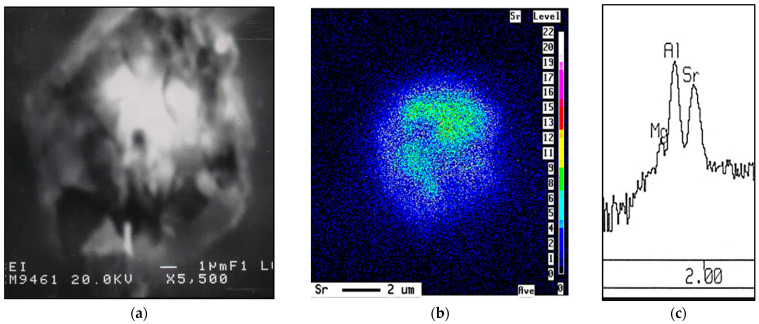
Formation of porosity in Sr-treated base alloy (AS alloy): (**a**) SEM micrograph, (**b**) Sr distribution in (**a**), (**c**) EDS corresponding to (**b**). Solidification rate was about 0.8 °C/s.

Figure 2 displays the tensile properties of the five alloys studied in the non-modified and Sr-modified conditions. The results indicate that the addition of Sr may lead to negative effect on the alloy ductility depending on the percentage of porosity associated with the formation of SrO as shown in alloy AS in Figure 3. Thus, careful attention should be paid when casting Sr-modified alloys.

**Figure 4:**
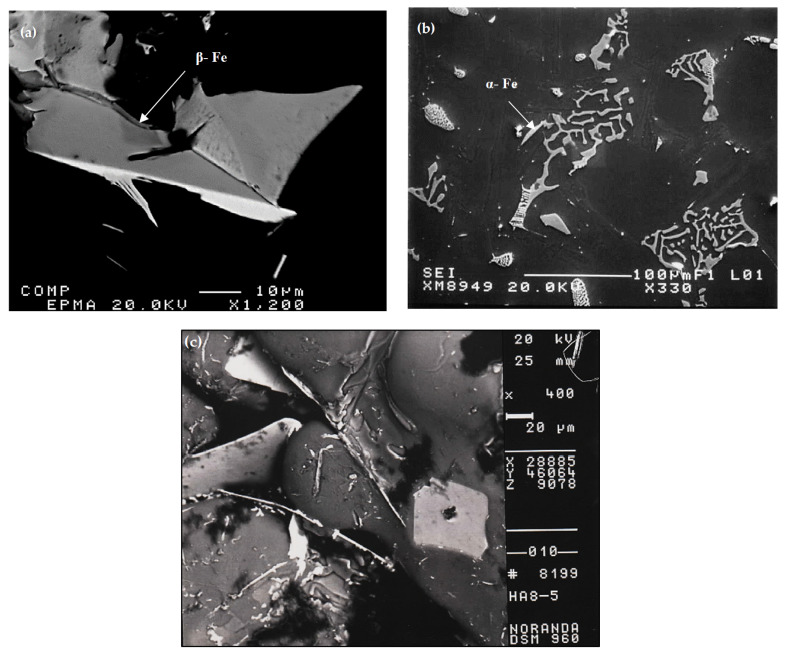
Backscattered electron images of (**a**) β-Al_5_FeSi, alloy A; (**b**) α-Al_15_(Fe, Mn)_3_Si_2_ phase, alloy AS; (**c**) porosity formed by β-platelets in alloy A. Solidification rate was about 0.8 °C/s.

In addition, Sr was found to alter the Fe-based intermetallic phase formed and its morphology, from the β-Fe phase to the α-Fe phase, as shown in Figure 4. The non-modified tensile bars that were solution-heat treated for 8 h/520 °C, quenched in warm water, and then aged for 8 h/175 °C, i.e., T6 tempered, gave an UTS value of ~285 MPa. Another problem encountered in examining the base alloy A was the formation of porosity caused by the precipitation of β-platelets during the course of solidification, which blocked the motion of the liquid metal as seen clearly in Figure 4c.

**Figure 5:**
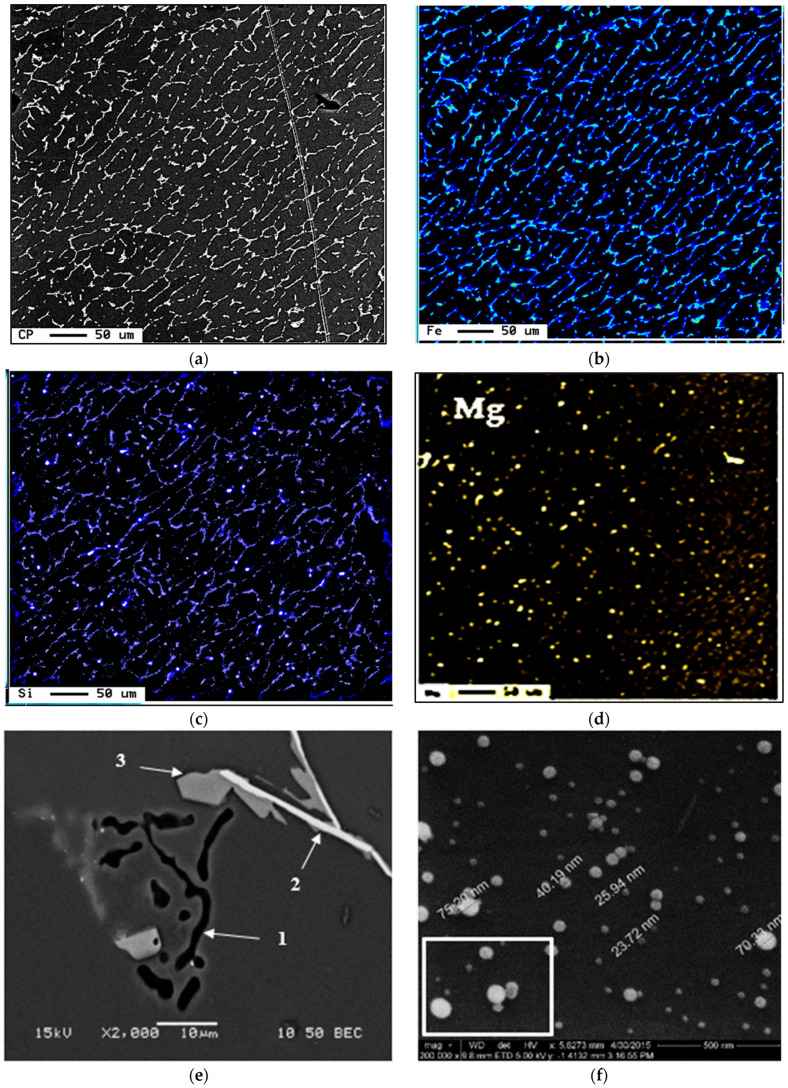

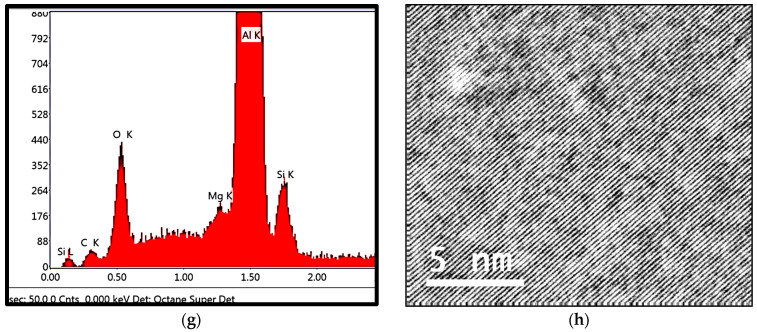
(**a**–**d**) Backscattered electron image of the as-received 6061 alloy, and X-ray images of Fe, Si and Mg (in that order)-note the difference in intensity of Fe and Si; (**e**) Backscattered electron images of 6061 alloy in as cast structure showing the presence of (1) Mg2Si, (2) β-Al5FeSi, (3) π-Al8FeMg3Si6, (**f**) precipitation of Mg2Si in T6 condition, (**g**) EDS spectrum corresponding to white square in (**b**) revealing reflections of Al, Si, and Mg elements, (**h**) high resolution bright field TEM electron image showing that the interplanar spacing is about 1.5-2Å.

Figure 5a–d illustrate the distribution of Al, Fe, Mg and Si in the as-received alloy after solution heat treatment using an electron probe microanalyzer. Figure 5e depicts the microstructure of the present 6061 alloy in the as-cast condition, revealing the co-existence of Mg_2_Si with β-Al_5_FeSi and π-Al_8_FeMg_3_Si_6_ phases. Following T6 heat treatment, a large number of fine Mg_2_Si precipitates were observed in the matrix with different sizes (2–70 nm). Some of these particles were agglomerated together as revealed in Figure 5f and confirmed by the associated EDS spectrum shown in Figure 5g.

**Figure 6:**
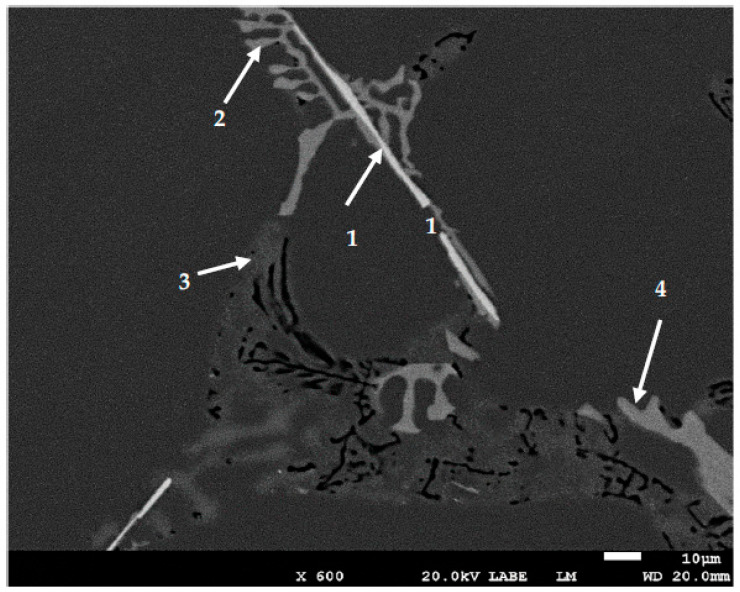
Backscattered electron image of alloy E in the as-cast condition. Solidification rate was about 8 °C/s.

**Figure 7:**
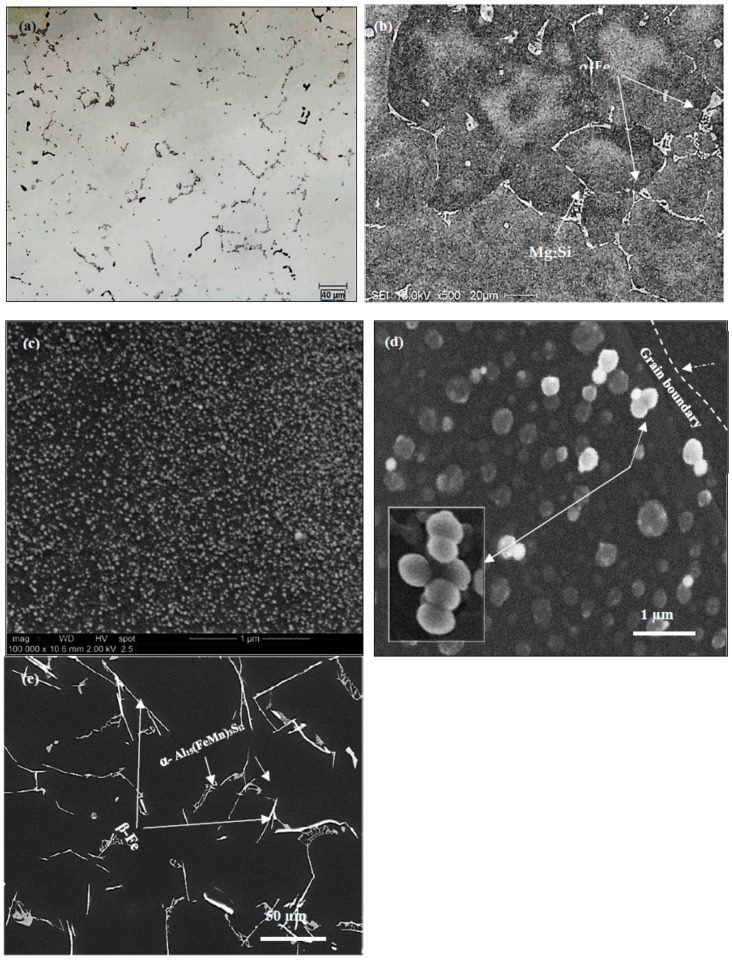
Precipitation of Mg_2_Si particles during aging of alloy B: (**a**) after T4 treatment; (**b**) after aging for 8 h at 175 °C—low magnification; (**c**) dense fine Mg_2_Si particles—after 8 h at 175 °C; (**d**) coarsening of Mg_2_Si—after 100 h at 175 °C; Note the accelerating voltage was 2 kV to demonstrate the sphericity of the particles as shown in the inset in (**d**); (**e**) fragmentation of β-platelets in BS alloy. Solidification rate was about 8 °C/s.

**Figure 8:**
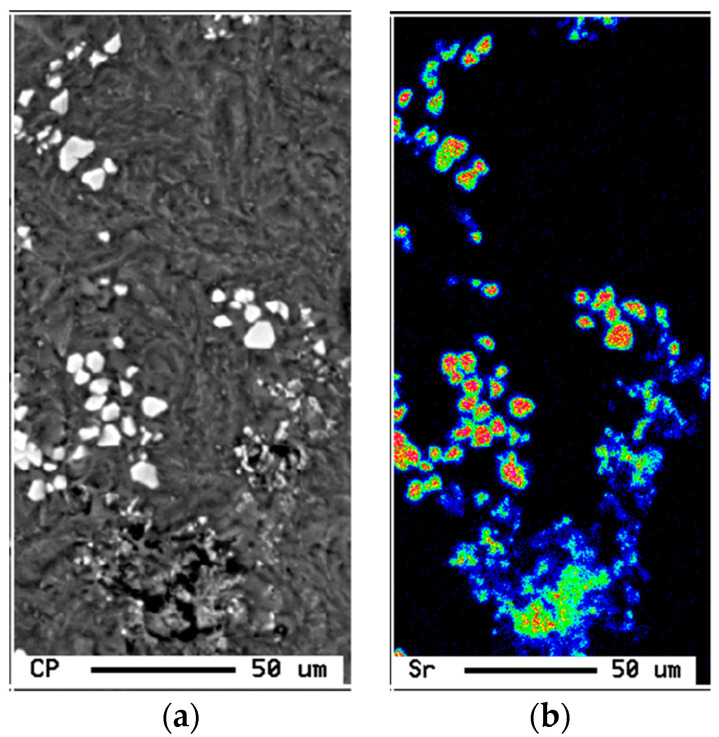
(**a**) Backscattered electron micrograph of CS alloy, and (**b**) corresponding distribution of Sr in the alloy. Solidification rate was about 0.8 °C/s.

As was seen in Figure 2, increasing the Si content to 1.49% in alloy A (i.e., alloy B), produced the highest UTS and YS levels compared to other alloys with % of elongation to fracture slightly lower than that exhibited by alloy A. Figure 6 reveals the microstructure of the alloy E in the as-solidified condition composed of small β-Fe platelets (marked 1) that were partially transformed to the π-Fe phase (marked 2), followed by precipitation of Mg_2_Si phase particles (marked 3), in addition to α-Fe particles (marked 4). The observed improvement in the alloy tensile properties may be attributed to the formation of the GP1 and GP2 zones as evident from the presence of multiple peaks and formation of a large volume fraction of Mg_2_Si particles as depicted in Figure 7. Modifying alloy B with 163 ppm Sr resulted in partial fragmentation of the β-Fe platelets, as shown in Figure 7e, leading to an increase in the UTS value by about 15 MPa—see Figure 2b. Figure 8 illustrates the Sr distribution in the CS alloy.

**Figure 9:**
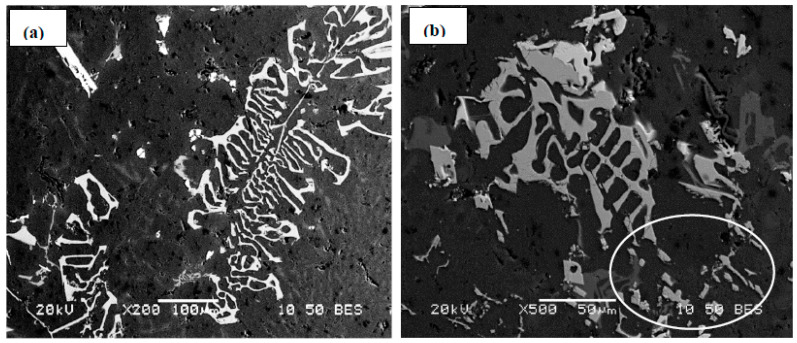
Shape and distribution of α-Fe phase particles in: (**a**) C alloy, (**b**) CS alloy. Solidification rate was about 0.8 °C/s. Note the fragmentation of the α-Fe particle (within the white circle) and its size in (**b**).

Increasing the Mn content in alloy A to 0.2% (i.e., alloy C) did not bring about much change in the alloy tensile parameters except when the alloy was modified by about 200 ppm Sr where the YS strength was seen to increase from 255 MPa (alloy C) to 277 MPa (alloy CS), at the cost of decreasing the maximum attainable ductility from 25% to 15%. The combined addition of Mn and Sr resulted in precipitation of the Fe-based intermetallics in the form of compacted Chinese script as shown in Figure 9a—at solidification rate of about 0.8 °C/s. According to Wang and. Xiong [23], fragmentation of the *α*-Al_15_(Fe, Mn)_3_Si_2_ phase takes place only at Sr concentrations on the order of ∼1500 ppm. As the concertation of Sr in the CS alloy is about 136 ppm, it is not expected to change the shape or size of the α-Fe particles as depicted in Figure 9b.

**Figure 10:**
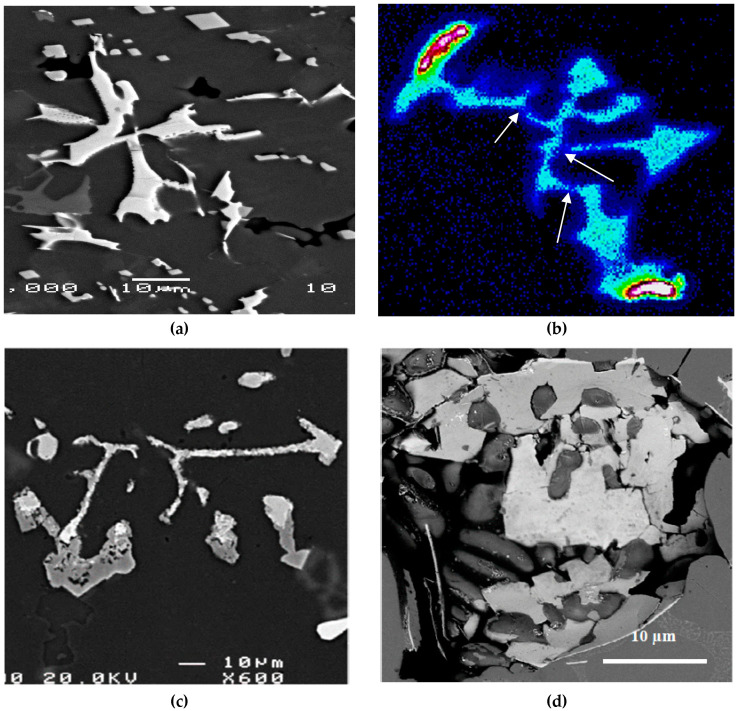
(**a**) Backscattered electron image of α-Fe in the as-cast condition for alloy DS; (**b**) Fe distribution in a α-Fe particle, (**c**,**d**) α-Al_8_Fe_2_SiBe—note the spongy nature of the Be-containing α-Fe. White arrows in (**b**) indicated fracture points. Solidification rate was about 0.8 °C/s.

An addition of about 200 ppm Be to the base alloy A (coded alloy D) enhanced the tensile properties in the T4 and T6 conditions by about 20–25 MPa (UTS is about 300 MPa after 24 h aging at 175 °C), at the cost of/reduction in the alloy ductility from 7.5% to 3.5% (almost 50%) and to 1.5% in the Sr-modified alloy DS. Additionally, modification of alloy D with about 170 ppm Sr resulted in fragmentation of α-Fe as revealed in Figure 10. Elsharkawi [24] reported on the possibility of the precipitation of α-Al_8_Fe_2_SiBe that is characterized by its rough surface as shown in Figure 10c. Figure 10d reveals the presence of a Be-containing α-Fe particle viewed within a pore, and taking the Chinese script form.

**Figure 11:**
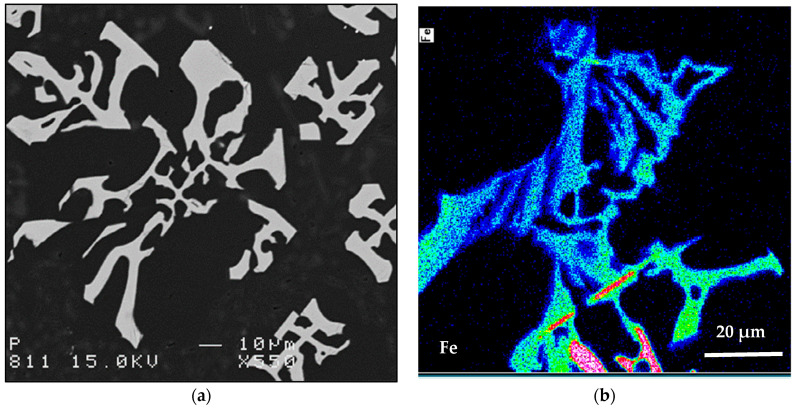
(**a**) Backscattered electron image of α-Fe in alloy ES, (**b**) Fe distribution in a α-Fe particle. Solidification rate was about 0.8 °C/s.

Increasing the Be addition to 400 ppm (alloy E) reduced the UTS by about 70 MPa in the T4 condition, more-or-less similar to the base alloy A. The E alloy also was characterized by clear formation of two peaks upon aging at 175 °C, at 3 and 24 h aging times. There is no marked difference in the aging behavior of alloy E modified with about 160 ppm Sr (ES alloy). For E and ES alloys, maximum % elongation to fracture was in the range of ~7 to 8% after 1 h of aging. Further aging resulted in a drop-in ductility to 1–2%. Figure 11 shows the effect of Be on the fragmentation of α-Fe. Thus, it is reasonable to conclude that Be leads to fragmentation of Fe-based intermetallics as well as changing the form of β-Fe platelets to a more spherical shape which improve the alloy tensile properties. It should be borne in mind, however, that the purpose of adding Be in the present work was not to promote its application but mainly to explore its effect.

**Figure 12:**
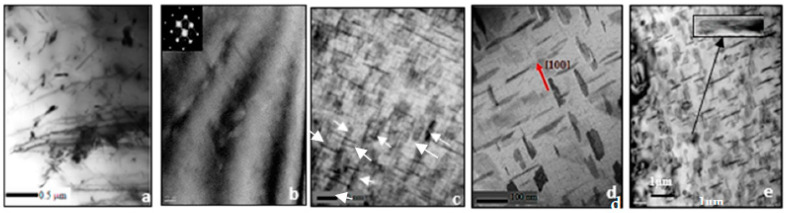
TEM micrographs corresponding to reactions shown in Table 2, after: (**a**) solution heat treatment at 495 °C/4 h; (**b**) aging for 2 h at 180 °C (GP zones + S′ − CuMgAl_2_); (**c**) aging for 6 h at 180 °C (high-density fine precipitates marked by white arrows); (**d**) aging for 24 h at 180 °C showing platelets of θ’-Al_2_Cu); (**e**) aging for 8 h at 240 °C, showing a mixture of θ’ (dark streaks) and θ-Al_2_Cu particles (pale phase in the inset micrograph) [25,26]. Reprinted with permission from Springer Nature, Inter Metalcast (2022) https://doi.org/10.1007/s40962-022-00824-7, accessed on 22 December 2022.

In general, decomposition of the supersaturated solution (SS) starts with the clustering of Si atoms. This leads to the formation of coherent spherical Guinier-Preston (GP) zones that elongate along the cube matrix direction to become needle-shaped (also referred to as β″ precipitates). The initially spherical GP zones (GP-1) convert to needle-like forms (GP-2) near the maximum strength inflections of the aging curves. As aging proceeds, the initially disordered zones become ordered and, with further aging, grow to form rods of an intermediate β′ phase, whose particles are semi-coherent with the matrix, with the rod axes parallel to the cube matrix directions. The final equilibrium Mg_2_Si phase (β-phase) forms as incoherent platelets on the aluminum matrix. Peak hardness is achieved before the platelets form [25,26,27,28,29,30,31]. According to the TEM work of Andrade et al. [25] and Tavitas-Medrano et al. [26], in the complex Al-Si Cu Mg system, with increasing aging temperatures, different decomposition and precipitation processes take place—in sequence in some cases, and simultaneously in others as summarized in Table 2 and Figure 12.

### 3.3. Q-Charts and ∆P

Drouzy et al. [32] proposed a Quality index *Q* to classify the quality of aluminum castings of Al-7%Si-0.3%Mg 356 alloy defined as:

(1) $$Q\;=\;\sigma_{UTS}\;+\;d\;\log\;(E_{f})$$

where *Q* is the quality index in MPa; $\sigma_{UTS}\;$ represents the ultimate tensile strength in MPa; *E_f_* is the percentage elongation to fracture; and *d* refers to a material constant equal to 150 MPa. The probable yield strength (σ*_P_*_(*YS)*)_ for the same alloy may be expressed as:

(2) $$\sigma_{P(YS)}\;=\;a\;\sigma_{UTS}\;-\;b\;\log\;(E_{f})\;+\;c$$

where the coefficients *a*, *b* and *c* for Al-7Si-Mg alloy were used as 1, 60 and −13, respectively, in MPa.

**Figure 13:**
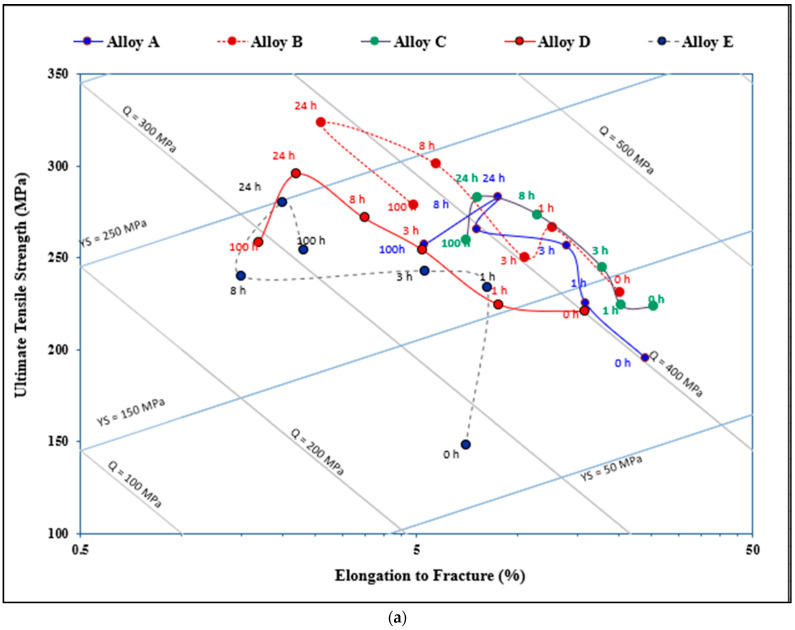

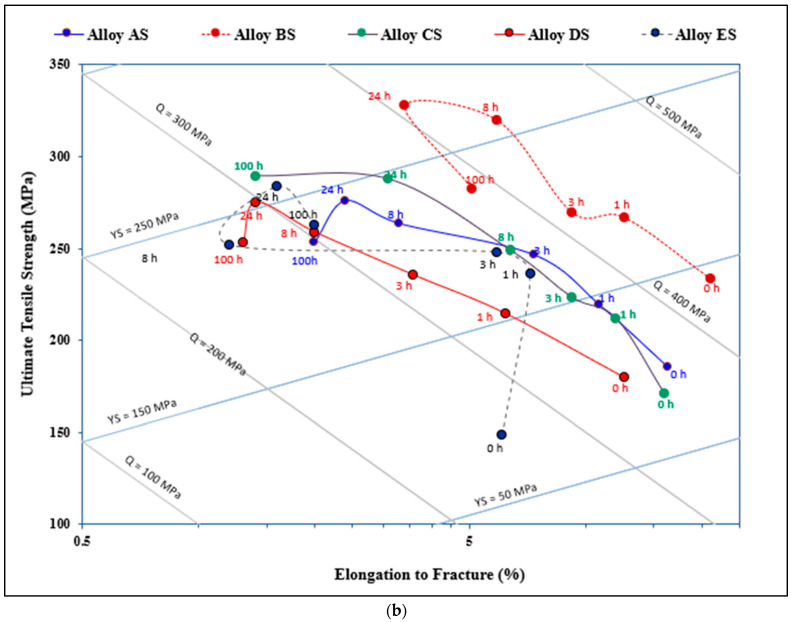
Q-charts of the present alloys: (**a**) non-modified, and (**b**) Sr- modified alloys.

Figure 13a depicts the effect of added alloying elements and aging conditions on the quality values of the present alloys. As can be observed, alloy A exhibits two peaks of more-or-less similar level of *Q* values (425 MPa) for aging times of 3 and 24 h, followed by a marked reduction to 365 MPa at 100 h, due to the marked decrease in the alloy strength, as shown in Figure 2. In the case of the high Si alloy (i.e., alloy B), the curve exhibits multiple peaks due the precipitation and coarsening of Mg_2_Si phase particles during the course of aging at 175 °C. The maximum attainable *Q* and UTS values are, respectively, 432 MPa (after 3 h aging) and 325 MPa (after 24 h aging). In the latter condition, however, the alloy achieved a low *Q* value of about 380 MPa after an aging time of 24 h. Further aging for times up to 100 h resulted only in reducing the UTS to about 280 MPa without noticeable change in the *Q*-level. Increasing the Mn concentration in the base alloy (i.e., alloy C), resulted in an almost steady-state curve at *Q* of about 430 MPa over a wide range of aging times (1–24 h), followed by a decrease to 385 MPa after 100 h of aging. The maximum attainable UTS was approximately 285 MPa (after 24 h aging time) which is very close to that of alloy A for the same aging time.

The Be-treated alloys demonstrated different behavior than the other alloys. Al low Be addition (about 200 ppm—alloy D), the curve is almost parallel to the *Q*-line during aging for 1–24 h at a value of 360 MPa, whereas the UTS levels increased from 224 to 295 MPa. Thus, the main reason for the marked drop in the alloy quality level is the continuous decease in the % elongation to fracture from ~10% to ~2.2%, respectively. Overaging of alloy D (100 h at 175 °C) leads to a noticeable decrease in both *Q* and UTS levels. Excessive increase in the Be concentration to 400 ppm resulted in the formation of two peaks: one after 1 h (*Q* = 370 MPa, UTS = 230 MPa), and the second after 24 h (*Q* = 325 MPa, UTS = 280 MPa), with a deep valley in between at 8 h (*Q* = 265 MPa, UTS = 240 MPa). A part of this dramatic variation in the alloy behavior may be attributed to the formation of porosity, as was shown in Figure 10d. It has been stated that Mg is more electropositive than the amphoteric Be and thus reacts more readily with most of the non-metals [32].

Figure 13b illustrates the effect of Sr addition (~200 ppm) on the behavior of the present alloys. It is evident that alloy B is the best of the five alloys in terms of maximum *Q* and UTS levels (*Q* = 430 MPa, UTS = 319 MPa) compared to the base alloy A (*Q* = 245 MPa, UTS = 275 MPa) under the same aging conditions. On the other hand, the BS alloy revealed the lowest *Q* and UTS values (*Q* = 330 MPa, UTS = 275 MPa). Considering the Be alloy (ES alloy), Sr did not exert a significant change in the alloy behavior in the sense that there is a large difference between the peak formed after 1 h aging time (*Q* = 365 MPa, UTS = 235 MPa) and the valley seen after 24 h (*Q* = 315 MPa, UTS = 285 MPa). Another point to be mentioned is that in both the non-modified and Sr- modified alloys, there is a marked increase in both the *Q* and UTS values by 100 MPa and 90 MPa, respectively, after the alloy was aged for only 1 h.

**Figure 14:**
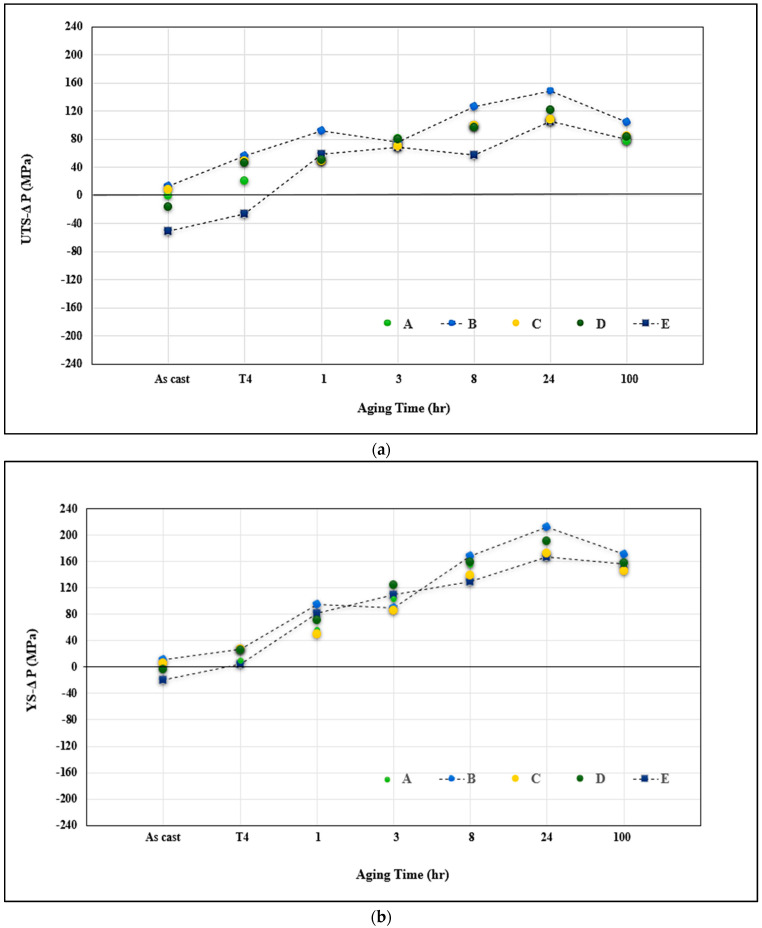

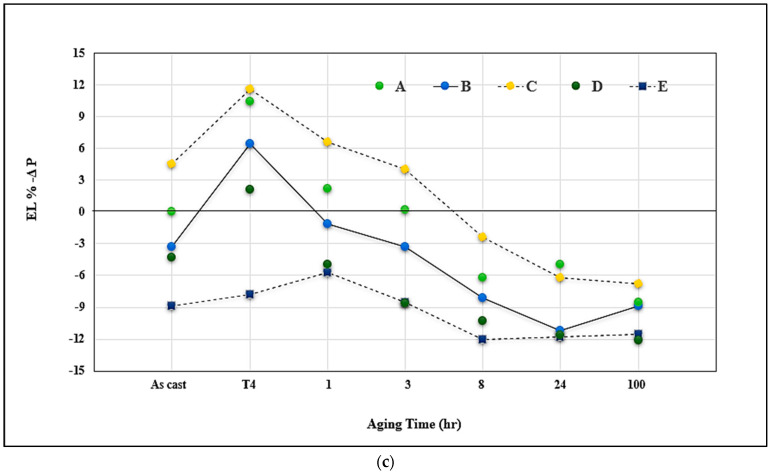
Effect of additives and aging times on the contribution to alloy A in the as-cast condition: (**a**) UTS, (**b**) YS, (**c**) % elongation to fracture for non-modified alloys.

The plots in Figure 14 summarize the contribution of both the additives and aging time at 175 °C. The data is presented in terms of ∆P which represents the difference in a specific property P of an alloy (i.e., UTS, YS or %El) at a given condition and that of alloy A in the as-cast condition, the latter being taken as the reference line for the calculation of all ∆P values shown in Figure 14. Figure 14a reveals that Si has a positive impact on the alloy UTS at all aging times, reaching a maximum after 24 h aging time (approximately 150 MPa). In contrast, alloy E offers the lowest contribution to the alloy A in the as-cast condition (−50 MPa in the as-cast case) and 108 MPa after 24 h aging. Contributions from the other alloys fall between these two alloys. In the T7 temper, all alloys showed a tendency for softening. Analysis of contribution to YS shows that the results obtained from the five studied alloys fall in a narrow band of approximately 20–30 MPa. Nevertheless, alloy B significantly improved the YS value of the as-cast base alloy A by about 210 MPa at the maximum hardening peak for all alloys (24 h at 175 °C) vs. 165 MPa offered by alloy E—Figure 14b. A wide dispersed pattern was the main observation for the effect of additives on the alloy ductility, as shown in Figure 14c. In general, precipitation of most of the Fe-based intermetallics in the form of spherical α-Fe particles registered the maximum contribution to the ductility of the base alloy whereas alloy E represented the minimum contribution (about −12% at the peak-aging condition). In this case, alloy B falls halfway between alloys C and E.

**Figure 15:**
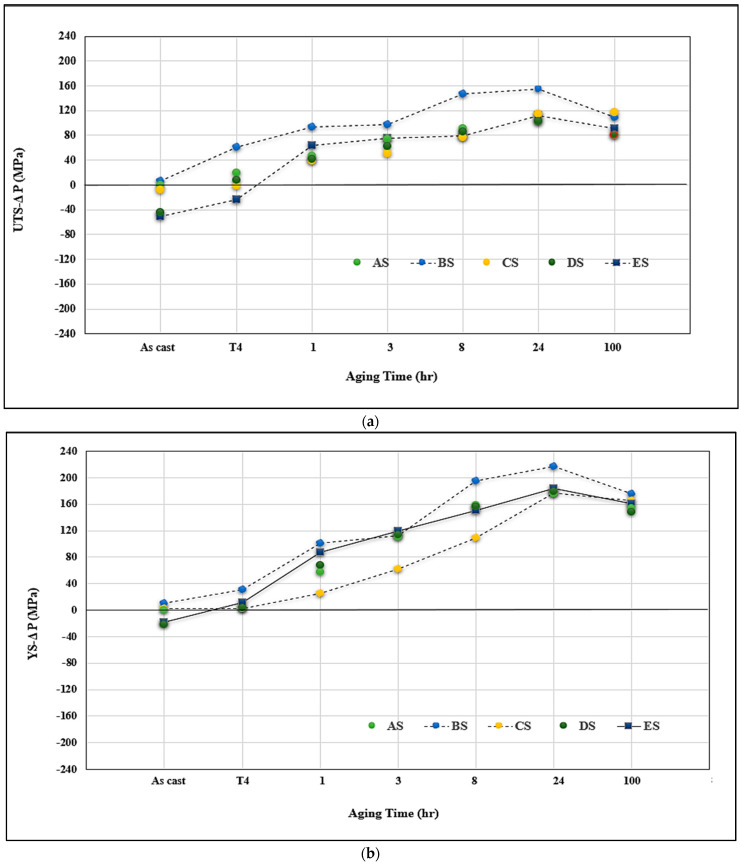

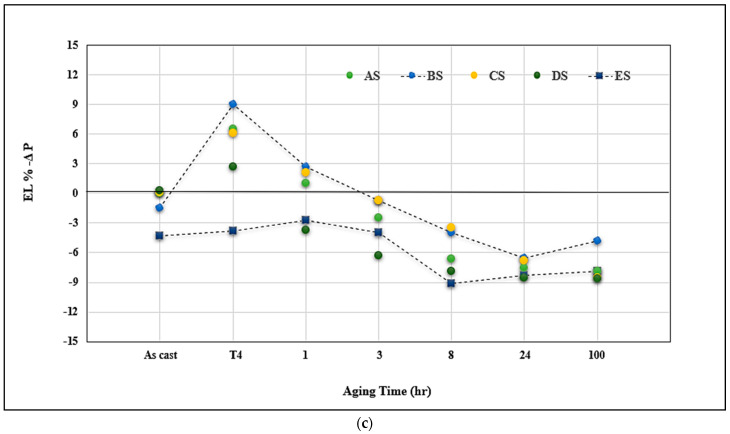
Effect of additives and aging times on contribution to alloy A in the as-cast condition: (**a**) UTS, (**b**) YS, (**c**) %elongation to fracture for the Sr-modified alloys.

Figure 15 evaluates the ∆P contribution of Sr addition in the Sr-modified alloys similar to that carried out for the non-modified alloys. In general, it is observed that the modified alloys followed the same pattern as that of non-modified ones. Examining the variation in the UTS values, the contribution from alloy BS is still the highest at the peak-aging condition. It is observed that the ∆P values for alloys CS and ES overlap at several aging times, which may be caused by the fragmentation of the α-Fe phase particles, as depicted in Figure 9 and Figure 11. The situation with ∆YS in the Sr-treated alloys is markedly different than that observed for the non-modified alloys where alloy ES revealed a superior contribution, above that shown by alloy CS. This may be explained in terms of the higher effectiveness of (Be + Sr) in fragmenting the α-Fe particles than the (Mn + Sr) addition as demonstrated in Figure 9 and Figure 11. Another point to be emphasized is that in the case of the Sr-modified alloys, the dispersion in the ductility values is lesser, as illustrated in Figure 15c, compared to what is observed in Figure 14c for the non-modified alloys.

### 3.4. Fractography

**Figure 16:**
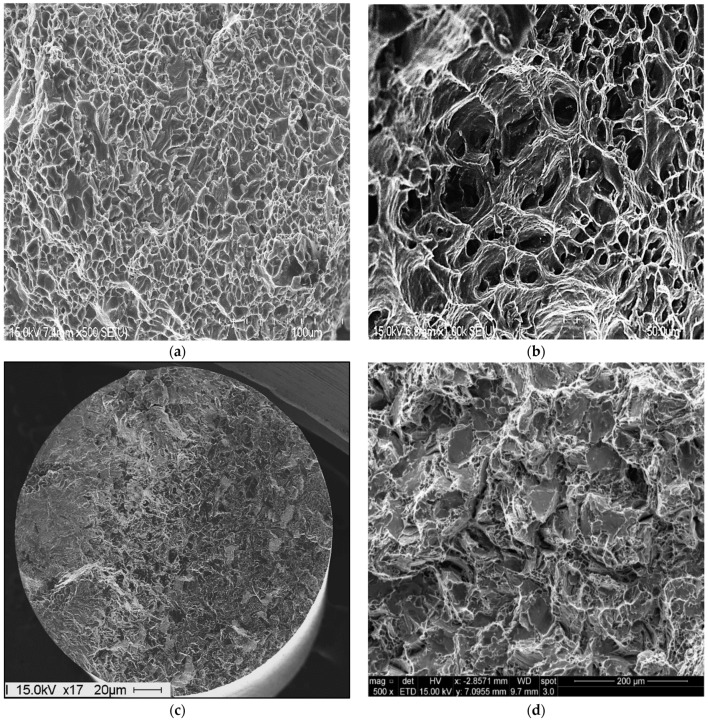
Fracture behavior of alloy A under different working conditions: (**a**) as-cast, (**b**) T4, (**c**) T6-low magnification, (**d**) same as in (**c**) taken at high magnification.

In the present section, the fracture behavior of the tensile bars of the present alloys will be limited to a discussion of alloy A and alloy ES to emphasize the role of Be, Sr and aging treatment. Figure 16 illustrates the fracture characteristics of alloy A. Figure 16a shows the fracture surface of the alloy in the as-cast condition, revealing a network of fine, well distributed dimples. With the increase in the alloy ductility after the solution heat treatment (8 h/520 °C) to about 25%, the fracture surface revealed a coarse dimple structure with a large number of slip lines covering the surfaces of the dimples, as depicted in Figure 16b. Aging alloy A at 175 °C for 24 h (peak aging) resulted in a drop in the % elongation to fracture to below 5%. Such marked decrease in the alloy ductility resulted in a fracture surface composed of fine dimples and cleavage throughout the matrix, as exhibited in Figure 16c. Figure 16d is a high magnification image of Figure 16c which shows the cleavage surface clearly.

**Figure 17:**
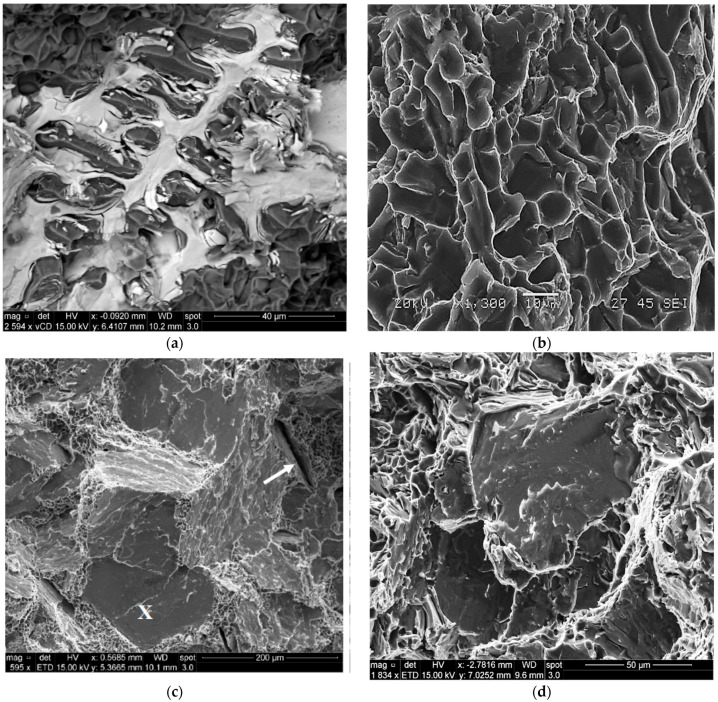
Fracture behavior of alloy ES under differed working conditions: (**a**) as-cast, (**b**) T4, (**c**) T6-low magnification, (**d**) same as in (**c**) high magnification.

As mentioned previously, the effect of simultaneous additions of Be and Sr caused fragmentation of the α-Fe particles, as inferred from the fracture surface of alloy ES in the as-cast condition displayed in Figure 17a. As in the case of alloy A, the fracture surface of the T4-treated tensile bar (7% El) consisted mainly of a coarse dimple structure as shown in Figure 17b, where the dimples are relatively smaller and less deep compared to that obtained from alloy A seen in Figure 16b. With further decease in the alloy ductility, reaching to about 1.5% at peak aging (24 h/175 °C), the fracture was predominantly cleavage type as presented in Figure 17c with some cracks (see white arrow). Figure 17d is a high magnification image of the area marked X in Figure 17c, illustrating details of the fractured particles observed in this area.

## 4. Conclusions

This is a comprehensive study on materials development providing various experimental data—from structure to property. It should be interesting to those who aim to find a solution for developing new engineering alloys, especially aluminum alloys, through a consideration of optimizing the combined effect of alloying elements and proper heat treatment as described in this work.

1. The main parameter controlling the type of Fe-based intermetallic phases which will precipitate in 6061 alloys is the Si/Fe ratio in such a way that a ratio of 1 will produce mainly the β-Al_5_FeSi phase, while a ratio of 2 will mainly result in α-Al_15_(Fe, Mn)_3_Si_2_ particles. Increasing the solidification rate encourages the precipitation of α-Al_15_(Fe, Mn)_3_Si_2_.
2. Although 6061 alloy does not contain a sufficient amount of Si to form the Al-Si eutectic, the addition of Sr in the order of 200 ppm leads to fragmentation of Fe-based intermetallics, particularly during solution heat treatment.
3. Addition of Be in amounts of 200–400 ppm results in:
  (a) fragmentation of α-Al_15_(Fe, Mn)_3_Si_2_ phase particles during solidification;
  (b) precipitation of a new compound, α-Al_8_Fe_2_SiBe in the form of Chinese script, characterized by its spongy surfaces instead of the smooth surfaces of the α-Al_15_(Fe, Mn)_3_Si_2_ phase;
  (c) possibility of porosity formation when Be is added in excess of 400 ppm.
4. Increasing the Si from 0.8% (in the base alloy) to 1.5% increases the volume fraction of the precipitated Mg_2_Si phase particles coupled with a marked improvement in the alloy strength. In all cases, the UTS curve reveals two peaks corresponding to the formation of GP1 and GP2 zones.
5. The combined addition of Si and Sr to the base alloy resulted in the best quality among the five studied alloys, whereas the addition of high Be (400 ppm) or Be + Sr produced an unpredictable behavior of the alloy quality.
6. Alloys containing high Si (without or with Sr) revealed the highest contribution to the strength of the base alloy with continuous increase up to the peak-aging condition. In contrast, high Be-containing alloys revealed the lowest contribution.

## Figures and Tables

**Table 1:** Chemical compositions of the alloys used in the present study (wt%).

Alloy	Alloying Elements (wt%)													
	Si	Fe	Cu	Mn	Mg	Cr	Ni	Zn	B	Be	Sr	V	Ti	Al
A	0.81	0.38	0.003	0.004	0.844	0.003	<0.005	0.013	0.003	0	0.0009	0.004	0.018	Bal.
AS	0.83	0.38	0.003	0.004	0.853	0.003	<0.005	0.013	0.003	0	0.0144	0.004	0.017	Bal.
B	1.49	0.35	0.003	0.006	0.682	0.002	<0.005	0.013	0.003	0	0.0014	0.004	0.018	Bal.
BS	1.49	0.334	0.002	0.004	0.859	0.002	<0.006	0.013	0.003	0	0.0163	0.004	0.019	Bal.
C	0.82	0.34	0.002	0.198	0.806	0.003	<0.005	0.013	0.003	0	0.0002	0.004	0.017	Bal.
CS	0.88	0.34	0.002	0.217	0.838	0.004	0.005	0.013	0.003	0	0.0206	0.005	0.015	Bal.
D	0.84	0.35	0.005	0.006	0.817	0.006	0.006	0.014	0.003	0.019	0.0038	0.007	0.016	Bal.
DS	0.84	0.34	0.003	0.005	0.806	0.004	<0.005	0.014	0.003	0.018	0.0147	0.007	0.019	Bal.
E	0.88	0.39	0.002	0.004	0.883	0.001	<0.005	0.013	0.004	0.040	0.0005	0.005	0.016	Bal.
ES	0.87	0.39	0.003	0.005	0.597	0.009	0.006	0.013	0.003	0.040	0.0182	0.005	0.017	Bal.

**Table 2:** Reactions taking place during solidification of Al-Si-Cu-Mg based alloys [25].

Stage	Heat Treatment	Phases
I	None	Mostly precipitated as equilibrium CuAl_2_ phases
II	Solution treatment and rapid cooling	All Cu in solution Most Mg phases in solution
III	Natural aging at room temperature	Segregation into GP I zones (coherent)
IV	Age hardening at 180 C	Dissolution of GP I zones Segregation into GP II zones (coherent) Precipitation of S’phase (CuAl_2_Mg)
	Further age hardening at 180 C	Increased diffusion into GP II zones and precipitation as θ phase (partially coherent) Precipitation of S′ phase (CuAl_2_Mg) Precipitation of β″ phase Precipitation of traces of Si
V	Overaging resulting from treatment time too long	Precipitated as θ phase, the equilibrium phase (incoherent)

## Data Availability

Data will be made available upon request.
